# Prognostic significance of Traf2- and Nck- interacting kinase (TNIK) in colorectal cancer

**DOI:** 10.1186/s12885-015-1783-y

**Published:** 2015-10-24

**Authors:** Hidenori Takahashi, Toshiaki Ishikawa, Megumi Ishiguro, Satoshi Okazaki, Kaoru Mogushi, Hirotoshi Kobayashi, Satoru Iida, Hiroshi Mizushima, Hiroshi Tanaka, Hiroyuki Uetake, Kenichi Sugihara

**Affiliations:** 1 Department of Surgical Oncology, Graduate School, Tokyo Medical and Dental University, 1-5-45 Yushima, Bunkyo-ku, Tokyo 113-8519 Japan; 2 Department of Translational Oncology, Graduate School, Tokyo Medical and Dental University, 1-5-45 Yushima, Bunkyo-ku, Tokyo Japan; 3 Department of Systems Biology, Graduate School of Biochemical Science, Tokyo Medical and Dental University, 1-5-45 Yushima, Bunkyo-ku, Tokyo Japan; 4 Center for Minimally Invasive Surgery, Division of Colorectal Surgery, Tokyo Medical and Dental University, 1-5-45 Yushima, Bunkyo-ku, Tokyo 113-8519 Japan; 5 Center for Public Health Informatics, National Institute of Public Health, 2-3-6 Minami, Wako-shi, Saitama 351-0197 Japan

**Keywords:** Colorectal cancer, Biomarker, Microarray analysis, Traf2- and Nck- interacting kinase (*TNIK*), Prognostic factor

## Abstract

**Background:**

The potential of expression profiling using microarray analysis as a tool to predict the prognosis for different types of cancer has been realized. This study aimed to identify a novel biomarker for colorectal cancer (CRC).

**Methods:**

The expression profiles of cancer cells in 152 patients with stage I-III CRC were examined using microarray analysis. High expression in CRC cells, especially in patients with distant recurrences, was a prerequisite to select candidate genes. Thus, we identified seventeen candidate genes, and selected Traf2- and Nck-interacting kinase (*TNIK*), which was known to be associated with progression in CRC through Wnt signaling pathways. We analyzed the protein expression of TNIK using immunohistochemistry (IHC) and investigated the relationship between protein expression and patient characteristics in 220 stage I-III CRC patients.

**Results:**

Relapse-free survival was significantly worse in the TNIK high expression group than in the TNIK low expression group in stage II (*p* = 0.028) and stage III (*p* = 0.006) patients. In multivariate analysis, high TNIK expression was identified as a significant independent risk factor of distant recurrence in stage III patients.

**Conclusion:**

This study is the first to demonstrate the prognostic significance of intratumoral TNIK protein expression in clinical tissue samples of CRC, in that high expression of TNIK protein in primary tumors was associated with distant recurrence in stage II and III CRC patients. This TNIK IHC study might contribute to practical decision-making in the treatment of these patients.

**Electronic supplementary material:**

The online version of this article (doi:10.1186/s12885-015-1783-y) contains supplementary material, which is available to authorized users.

## Background

Colorectal cancer (CRC) is one of the most common cancers worldwide, and the third leading cause of cancer death in Japan [1]. Although curative surgery is performed, approximately 30 % of CRC patients develop recurrences [2]. In particular, recurrences in distant organs or distant lymph nodes (distant recurrences) have a critical impact on the prognosis of CRC.

The TNM classification system of the International Union Against Cancer (UICC) has been used as a standard tool for prognostic stratification and has provided valuable information for therapeutic decision-making, such as the selection of suitable candidates for adjuvant chemotherapy. However, TNM classification is limited in its ability to predict the exact prognosis of individual CRC patients. For further improvement in the prognosis for CRC patients and individualization of cancer therapy, molecular biological approaches have been intensely studied recently.

Expression profiling using microarray analysis allows the exploration of several thousand cancer-related or cancer-specific genes in the search for candidate genes amenable to prognosis prediction and classification of human malignancies [3–5]. A number of genes that were poor prognostic markers have been identified in CRC patients (e.g., *NUCKS1*, *BNIP3*, *PDGFC*, *OPG*) using microarray analysis; their prognostic significance was subsequently assessed in surgically resected CRC subjects by mRNA and/or immunohistochemical (IHC) analyses [6–9].

In this study, we identified Traf2- and Nck-interacting kinase (*TNIK*) using microarray analysis as a candidate gene that was related to distant recurrence of CRC. *TNIK* is a member of the germinal center kinase family, and is reported to be essential for Wnt signaling and CRC proliferation and progression [10, 11]. However, the correlation between intratumoral TNIK expression and the prognosis of CRC patients has not yet been reported. This study aimed to confirm the prognostic significance of TNIK in CRC through investigation of the relationship between intratumoral TNIK protein expression and the prognosis of CRC patients.

## Methods

### Patients

Primary tumors from 1210 consecutive patients who underwent surgical resection for CRC between 2002 and 2009 at Tokyo Medical and Dental University Hospital were included in this study. This study was conducted in accordance with the Declaration of Helsinki and Ethical Guidelines for Epidemiological Study published by the Japanese government. The Ethical Committee of Tokyo Medical and Dental University Hospital approved the study, and written informed consent was obtained from all patients.

A total of 152 patients were assigned to the microarray analysis, including 27 patients with stage I, 69 patients with stage II, 56 patients with stage III disease. Distant recurrences (not including local recurrences) occurred in 26 patients. Patient characteristics of these subjects for microarray analysis were shown in Additional file 1. The median follow-up time at the analysis was 60 months (range 15–76 months).

For the protein expression analysis using immunohistochemistry (IHC), the different 220 patients were studied; 48, 87, and 85 patients with stage I, II, and III CRC, respectively. Distant recurrences occurred in 53 patients. The median follow-up time at the analysis was 61 months (range 1–141 months).

### RNA extraction

Cancer tissues were immediately embedded in Tissue-Tek OCT compound (Sakura Finetek Japan, Tokyo, Japan) after surgical resection. Serial frozen sections of 9-μm thickness were mounted onto a foil-coated glass slide, 90 FOIL-SL25 (Leica Microsystems, Wetzlar, Germany), and laser capture microdissection (LCM) was carried out using the Application Solutions Laser Capture Microdissection System (Leica Microsystems). Total RNA from LCM of cancer tissues and normal colorectal epithelia were extracted using the RNeasy mini Kit (QIAGEN, Hilden, Germany) according to the manufacturer’s protocol. The integrity of the obtained total RNA was assessed using an Agilent 2100 BioAnalyzer (Agilent Technologies, Palo Alto, CA), and samples with an RNA integrity number greater than 5.0 were used for microarray analysis.

### Oligonucleotide microarray analysis

cRNA was prepared from 100 ng total RNA using two-cycle target labeling and a control reagents kit (Affymetrix, Santa Clara, CA). Hybridization and signal detection of the Human Genome U133 Plus 2.0 arrays (Affymetrix) were carried out according to the manufacturer’s instructions. The gene expression data was deposited in the Gene Expression Omnibus (GEO) under accession ID GSE71222 (http://www.ncbi.nlm.nih.gov/geo/query/acc.cgi?token=izcnsaygjjkpped&acc=GSE71222).

The gene expression data were then analyzed to identify the candidate genes related to distant recurrence. We defined the patients with distant recurrence as the recurrence group, and the patients without any distant recurrences as the non-recurrence group. The criteria to select candidate genes were as follows; (i) a higher expression level in cancer tissues than in non-cancerous mucosa, (ii) a significantly higher expression level in cancer cells of the recurrence group than in the non-recurrence group. A higher expression was defined as a numerical value 1.5 times greater than those in another group.

### Immunohistochemistry

A streptavidin-biotin method was used for TNIK immunostaining. Formalin-fixed paraffin-embedded tissue blocks from each patient were cut at 4-μm thickness. After deparaffinization in xylene and rehydration through a series of incubations in decreasing concentrations of ethanol, antigens were retrieved from the tissues by autoclaving them at 121 °C for 2 min in pH 8.0 citrate buffer. The sections were then incubated in a solution of 3 % hydrogen peroxide in 100 % methanol for 15 min at room temperature in order to quench endogenous peroxidase activity. Nonspecific binding was blocked by treating the tissues with 10 % normal goat serum (Nichirei Bioscience, Tokyo, Japan) for 15 min. Thereafter, the slides were incubated with a rabbit polyclonal antibody against TNIK at a 1:400 dilution (HPA012128; Sigma-Aldrich, Co. LLC., St Louis, MO) for 30 min at room temperature and for overnight at 4 °C. Next, sections were incubated with labeled polymer [Histofine® Simple Stain MAX PO (MULTI); Nichirei Bioscience] for 30 min at room temperature. Color development was carried out with DAB (0.02 % 3, 30-diaminobenzidine tetrahydrochloride; Nichirei Bioscience) for 7 min at room temperature. The slides were counterstained with 1 % Mayer’s hematoxylin, after which they were dehydrated using a series of increasing alcohol concentrations, immersed in xylene, and finally coverslipped.

All sections were scored by two investigators. The staining intensity was scored as 0 (negative), 1 (weak), 2 (moderate), or 3 (strong). The staining extensity was scored as 1 (0–10 % of the tumor cells stained), 2 (11–40 %), 3 (41–70 %), or 4 (71–100 %). The sum of the intensity and extensity scores, potentially ranging from 1 to 7, was calculated, and the average of three fields (magnification 100×) that were strongly stained at the tumor front were used to determine the TNIK IHC-staining score for each patient.

### Statistical analysis

Statistical analyses were carried out using SPSS version 17.0 software for Windows (SPSS Inc, Chicago, IL). To estimate the significance of differences between the groups, the Wilcoxon signed-rank test, Mann–Whitney *U* test, and *χ*^2^ test were used where appropriate. Relapse-free survival (RFS) was defined as the time from the date of surgery to distant recurrence. Overall survival (OS) was calculated from the date of surgery to death from any cause. Survival curves were estimated using the Kaplan–Meier method, and curves were compared using the log-rank test. Factors affecting RFS and OS were examined with univariate and multivariate analyses using the Cox proportional hazards model. A p value < 0.05 was considered statistically significant.

## Results

### Microarray analysis

In the microarray analysis, 69 genes were identified that fulfilled the above-mentioned criteria. The heat map of 69 genes is shown in Additional file 2. Among 69 genes, 17 genes were reported to be associated with human malignancies. The list of selected 17 genes is shown in Additional file 3. Among 17 candidate genes, we focused on 5 genes (*S100A2, TNIK, TESC, PROX1* and *ZBED6*) which reported to be associated with colorectal cancer. Another researcher in our laboratory is submitting a paper about *S100A2. TESC, PROX1* and *ZBED6* are being researched now. We selected *TNIK* as a target gene for further analysis for the following reasons; (i) *TNIK* is known to be associated with the progression of CRC through Wnt signaling pathways [10, 11], (ii) *TNIK* gene amplification was reported to be required for the progression of gastric cancer, and nuclear expression of *TNIK* in hepatocellular carcinoma was reported to be associated with poor prognosis [12, 13], (iii) the correlation between the expression levels of TNIK in CRC patients and their clinical outcomes have not been revealed. Co-expression analysis of 69 genes was performed using STRING DATA BASE (http://string-db.org/). The co-expression networks are shown in Additional file 4. Although some co-expression networks were observed, *TNIK* was not involved in those networks.

### Expression of TNIK protein in CRC

**Fig. 1:**
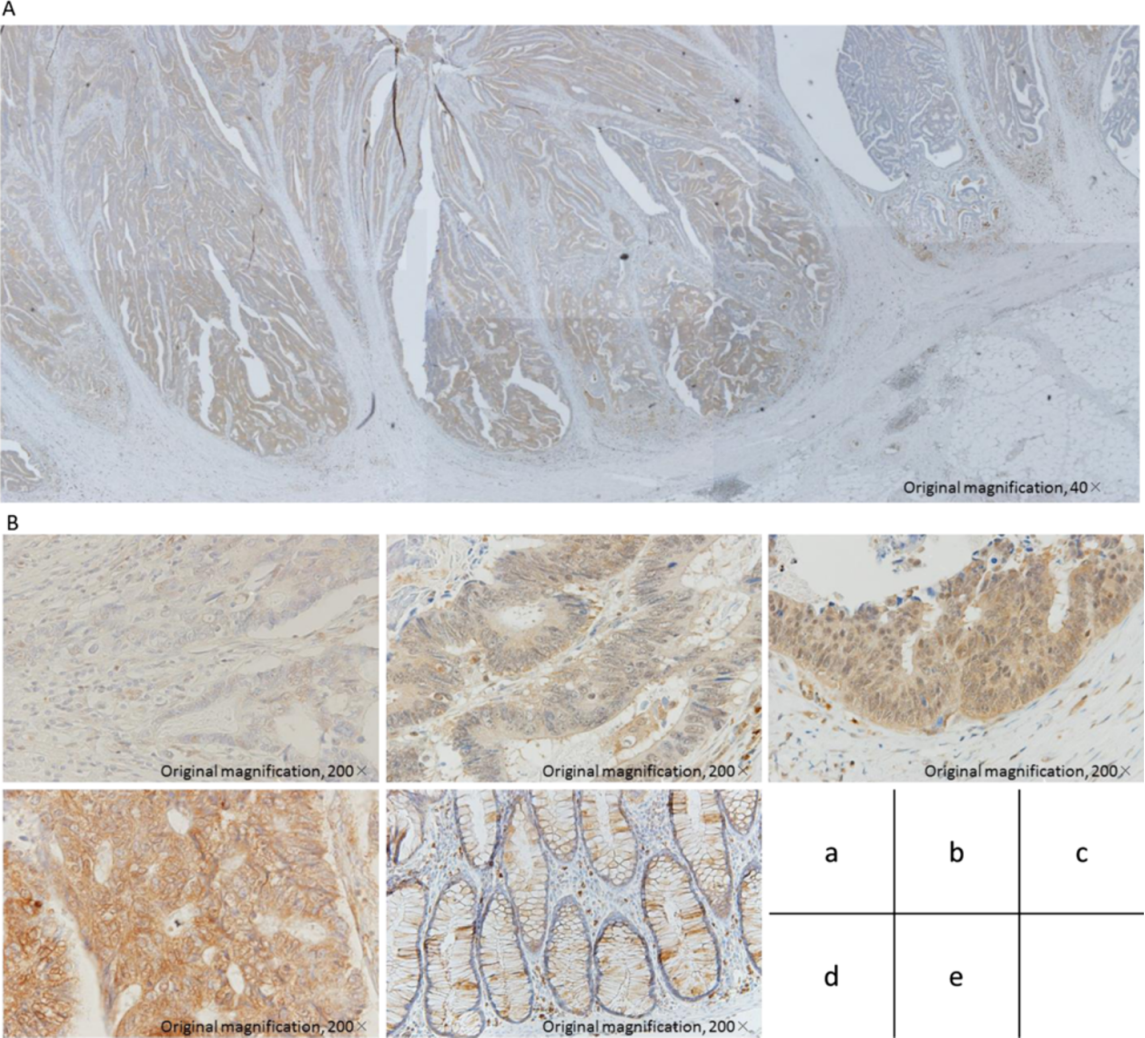
TNIK immunostaining in representative CRC and normal colorectal epithelium. **a** Examining the entire tumor cross-section revealed that TNIK staining was heterogeneous. **b** (*a*) 0 no staining; (*b*) 1+ weak staining; (*c*) 2+ moderate staining; (*d*) 3+ strong staining; (*e*) normal epithelium staining

Figure 1 shows the representative results of IHC staining of TNIK in CRC samples. TNIK protein was observed in the cytoplasm of cancer cells, but not in normal epithelial cells. The entire tumor cross-section showed heterogeneous staining; however, the staining tended to be strong at the invasive tumor front. Therefore, we focused on TNIK expression at the invasive tumor front, and used the staining scores at the invasive tumor front in further analysis.

### TNIK expression and patient characteristics

The median of the staining score of the 220 CRC patients studied was 5.3 (range 1-7). The scoring cut-off point was determined using receiver operating characteristic (ROC) curve analysis. This was conducted for TNIK staining scores in comparison with CRC distant recurrence. The experimental samples were divided into 2 groups by the cut-off score: the low expression group (staining score < 6, *n* = 139) and the high expression group (staining score ≥ 6, *n* = 81).

Relationships between TNIK expression and patient characteristics are shown in Table 1. T4, right-sided colon cancer, lymphatic invasion, and venous invasion were significantly associated with high expression of TNIK. Samples from patients who developed distant recurrences showed higher TNIK expression than those from patients without distant recurrence.

**Table 1:** Patient characteristics and TNIK expression in stage I-III CRC patients

Variables		TNIK expression		*p* value
		Low (*n* = 139)	High (*n* = 81)	
Age	< 70 years	98	53	0.435
	≥ 70 years	41	28	
Gender	male	88	60	0.102
	female	51	21	
Histology (TNM 7th)	G1	62	28	0.258
	G2	70	46	
	G3	7	7	
Tumor location	right-sided colon	26	28	0.016
	left-sided colon	58	22	
	rectum	55	31	
Stage (TNM 7th)	I	36	12	0.104
	II	55	32	
	III	48	37	
Depth of tumor invasion (TNM 7th)	T1	17	6	T1-3 vs. T4 0.002
	T2	32	11	
	T3	58	29	
	T4	32	35	
Lymphatic invasion	(-)	50	17	0.021
	(+)	89	64	
Venous invasion	(-)	27	6	0.020
	(+)	112	75	
Lymph node metastasis	(-)	91	44	0.102
	(+)	48	37	
CEA*^1^	< 5 ng/ml	101	52	0.189
	≥ 5 ng/ml	38	29	
Distant recurrence	(-)	118	49	< 0.001
	(+)	21	32	

CEA*^1^ carcinoembryonic antigen

### TNIK expression and prognosis of CRC patients

**Fig. 2:**
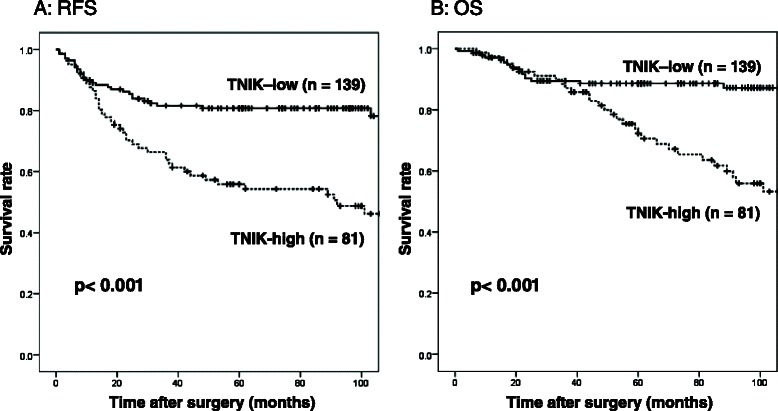
**a** RFS and (**b**) OS curves of 220 stage I-III CRC patients stratified by TNIK expression

Figure 2a and 2b show RFS and OS curves stratified by TNIK expression group. RFS as well as OS in the TNIK high expression group was significantly worse (*p* < 0.001 log-rank test) than in those in the TNIK low expression group.

In the univariate analysis, advanced age (≥70 years), poorly differentiated histology, T4, lymphatic invasion, venous invasion, lymph node metastasis, elevated carcinoembryonic antigen (CEA) level, and high TNIK expression were significantly associated with worse RFS. In the multivariate analysis, high TNIK expression, lymph node metastasis, and advanced age were identified as independent factors associated with worse RFS (Table 2).

**Table 2:** Univariate and multivariate analyses of clinicopathologic variables affecting A: RFS and B: OS in patients with stage I-III CRC

Variables		No. Patients	Univariate analysis		Multivariate analysis	
			Hazard ratio (95 % CI)	*p* value	Hazard ratio (95 % CI)	*p* value
≪RFS≫						
Age	< 70 years	151	0.614 (0.377–0.988)	0.049	1.849 (1.115–3.067)	0.017
	≥ 70 years	69				
Gender	male	148	1.709 (0.974–2.996)	0.062		
	female	72				
Histology (TNM 7th)	G1	90	0.485 (0.285–0.826)	0.008	1.555 (0.884–2.737)	0.125
	G2, G3	130				
Tumor location	colon	134	1.473 (0.911–2.382)	0.114		
	rectum	86				
Depth of tumor invasion (TNM 7th)	T1-3	153	2.713 (1.342–3.520)	0.002	1.061 (0.616–1.827)	0.832
	T4	67				
Lymphatic invasion	(-)	67	2.647 (1.386–5.054)	0.003	1.344 (0.643–2.809)	0.432
	(+)	153				
Venous invasion	(-)	33	3.260 (1.186–8.959)	0.022	1.334 (0.457–3.893)	0.598
	(+)	187				
Lymph node metastasis	(-)	135	3.316 (2.018–5.447)	< 0.001	2.326 (1.276–4.243)	0.006
	(+)	85				
CEA	< 5 ng/ml	153	2.239 (1.379–3.634)	0.001	1.498 (0.907–2.473)	0.114
	≥ 5 ng/ml	67				
TNIK expression	low	139	2.762 (1.694–4.502)	< 0.001	2.184 (1.310–3.641)	0.003
	high	81				
Variables		No. Patients	Univariate analysis		Multivariate analysis	
			Hazard ratio (95 % CI)	*p* value	Hazard ratio (95 % CI)	*p* value
≪OS≫						
Age	< 70 years	151	2.842 (1.589–5.083)	< 0.001	2.887 (1.559–5.343)	0.001
	≥ 70 years	69				
Gender	male	148	0.451 (0.218–0.936)	0.032	0.598 (0.281–1.272)	0.182
	female	72				
Histology (TNM 7th)	G1	90	2.046 (1.076–3.889)	0.029	1.572 (0.788–3.137)	0.199
	G2, G3	130				
Tumor location	colon	134	1.301 (0.726–2.332)	0.376		
	rectum	86				
Depth of tumor invasion (TNM 7th)	T1-3	153	3.047 (1.706–5.440)	< 0.001	1.693 (0.865–3.314)	0.124
	T4	67				
Lymphatic invasion	(-)	67	2.368 (1.104–5.077)	0.027	1.226 (0.517–2.909)	0.643
	(+)	153				
Venous invasion	(-)	33	4.483 (1.086–18.500)	0.038	1.709 (0.385–7.591)	0.481
	(+)	187				
Lymph node metastasis	(-)	135	2.582 (1.435–4.648)	0.002	1.505 (0.733–3.093)	0.266
	(+)	85				
CEA	< 5 ng/ml	153	2.608 (1.461–4.657)	0.001	1.692 (0.929–3.082)	0.086
	≥ 5 ng/ml	67				
TNIK expression	low	139	3.373 (1.838–6.192)	< 0.001	2.279 (1.205–4.310)	0.011
	high	81				

*CI* confidence interval

With respect to OS, univariate analysis indicated that advanced age, female gender, poorly differentiated histology, T4, lymphatic invasion, venous invasion, lymph node metastasis, elevated CEA level, and high TNIK expression were significantly associated with worse OS. High expression of TNIK and advanced age were identified as independent factors associated with worse OS by multivariate analysis (Table 2).

### TNIK expression and prognosis of stage II and III patients

**Fig. 3:**
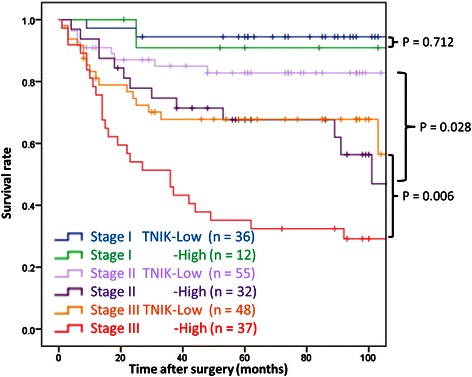
RFS curves stratified by TNM-7^th^ stage and TNIK expression

Figure 3 shows RFS curves stratified by TNM-7^th^ stage and TNIK expression group.

RFS curves were clearly separated by stage subgroup, and were also significantly separated by TNIK expression group in both stage II and III patients. RFS was significantly worse in the TNIK high expression group than in the TNIK low expression group in stage II (*p* = 0.028) and stage III (*p* = 0.006).

In the multivariate analysis, high TNIK expression was identified as a statistically significant and independent risk factor of distant recurrence in stage III patients, but not reached statistical significance in stage II (Table 3).

**Table 3:** Univariate and multivariate analyses of clinicopathologic variables affecting RFS in patients with A: stage II CRC and B: stage III CRC

Variables		No. Patients	Univariate analysis		Multivariate analysis	
			Hazard ratio (95 % CI)	*p* value	Hazard ratio (95 % CI)	*p* value
≪stage II≫						
Age	< 70 years	53	2.984 (1.249–7.130)	0.014	2.798 (1.168–6.702)	0.021
	≥ 70 years	34				
Gender	male	53	0.506 (0.198–1.294)	0.155		
	female	34				
Histology (TNM 7th)	G1	36	1.041 (0.445–2.438)	0.926		
	G2, G3	51				
Tumor location	colon	57	1.723 (0.744–3.989)	0.204		
	rectum	30				
Depth of tumor invasion (TNM 7th)	T1-3	62	1.269 (0.517–3.115)	0.603		
	T4	25				
Lymphatic invasion	(-)	36	1.292 (0.541–3.083)	0.564		
	(+)	51				
Venous invasion	(-)	11	1.550 (0.362–6.635)	0.555		
	(+)	76				
Examined lymph nodes	< 12	21	0.820 (0.321–2.097)	0.679		
	≥ 12	66				
CEA	< 5 ng/ml	62	1.153 (0.450–2.954)	0.767		
	≥ 5 ng/ml	25				
TNIK expression	low	55	2.511 (1.073–5.877)	0.034	2.323 (0.990–5.452)	0.053
	high	32				
Variables		No. Patients	Univariate analysis		Multivariate analysis	
			Hazard ratio (95 % CI)	*p* value	Hazard ratio (95 % CI)	*p* value
≪stage III≫						
Age	< 70 years	60	1.032 (0.536–1.988)	0.925		
	≥ 70 years	25				
Gender	male	60	0.681 (0.334–1.385)	0.289		
	female	25				
Histology (TNM 7th)	G1	24	1.838 (0.878–3.846)	0.106		
	G2, G3	61				
Tumor location	colon	48	1.540 (0.839–2.824)	0.163		
	rectum	37				
Depth of tumor invasion (TNM 7th)	T1-3	43	1.308	0.388		
	T4	42				
Lymphatic invasion	(-)	4		0.706		
	(+)	81				
Venous invasion	(-)	3		0.570		
	(+)	82				
Lymph node metastasis (TNM 7th)	N1	48		0.194		
	N2	37				
CEA	< 5 ng/ml	47	1.918 (1.042–3.530)	0.036	1.678 (0.903–3.120)	0.102
	≥ 5 ng/ml	38				
TNIK expression	low	48	2.345 (1.256–4.378)	0.007	2.141 (1.136–4.038)	0.019
	high	37				

## Discussion

This study is the first to demonstrate the prognostic significance of intratumoral TNIK protein expression in clinical tissue samples of CRC. Here, we found that there were some significant correlations between intratumoral TNIK protein expression and clinicopathologic features in CRC patients, with high TNIK expression being a significant prognostic factor of distant recurrence after curative surgery for stage II and III CRC.

*TNIK* is one of the germinal center kinase family members involved in cytoskeleton organization and neural dendrite extension [14–16], and is reported to be associated with the progression of several human cancers [12, 13]. Wnt signaling maintains the undifferentiated state of intestinal crypt cells through the T-cell for factor 4 (TCF4)/β-catenin-activating transcriptional complex. In CRC, activating mutations in Wnt pathway components lead to inappropriate activation of the TCF4/β-catenin transcriptional program and carcinogenesis [10]. Activation of the Wnt signaling cascade leads to disruption of the β-catenin degradation complex, resulting in β-catenin accumulation in the cytoplasm. β-catenin translocates to the nucleus, where it serves as a transcription factor to activate downstream target genes by binding to TCF4 [17–19]. TNIK is activated by β-catenin at the cytoplasm and translocates into the nucleus. Then, activated TNIK directly binds with both TCF4 and β-catenin and phosphorylates TCF4. The kinase activity of TNIK may be essential in TCF/lymphoid enhancer factor (LEF)-driven transcriptional activation of Wnt signaling pathways [11]. In the present study, high expression of TNIK was significantly associated with tumor depth (T4), lymphatic invasion, and venous invasion. This result suggests that elevated expression of TNIK may accelerate tumor progression and invasion.

We found that TNIK staining tended to be stronger at the invasive tumor front than at the tumor marginal site. The balance of pro- and anti-tumor factors at the invasive tumor front is reported to be decisive in determining tumor progression and the clinical outcome of patients with CRC [20, 21]. Our results supported the idea that high expression of TNIK at the invasive tumor front might play an important role in progression and distant metastasis of CRC.

Classification of risk of recurrence is intensively studied in the field of cancer treatment, because of its importance in treatment decision-making. Postoperative adjuvant chemotherapy for patients with stage III CRC is internationally accepted as a standard care for improving survival. de Gramont et al. indicated that stage III consists of subgroups of patients with various risks of recurrence, and proposed selecting treatment regimens from several options according to risk subgroup-specific survival data [22, 23]. On the basis of our results, stage III CRC patients with high TNIK expression may require strong adjuvant chemotherapy. For stage II disease, major western guidelines recommend adjuvant chemotherapy when patients have risk factors including T4 lesions, less than 12 lymph nodes examined, perforation, poorly differentiated histology, and lymphovascular involvement, even though the efficacy of adjuvant chemotherapy for stage II CRC has not been well-established and remains controversial [24]. Our results proposed that stage II CRC patients with high TNIK expression might be candidates for adjuvant chemotherapy as high-risk patients of distant recurrence.

Furthermore, the development of novel therapies that target critical biological pathways has greatly expanded treatment options for patients with CRC and resulted in substantial improvements in survival in the last decade [25, 26]. The Wnt signaling pathway has arisen as a target, and development of a new drugs targeting TNIK for CRC is in progress both in Japan and western country [27, 28].

## Conclusions

High expression of TNIK protein in primary tumors was associated with distant recurrence in stage II and III CRC patients. This TNIK IHC study might contribute to practical decision-making in treatment for these patients.

## Additional files

Additional file 1: **Patients’ characteristics for microarray analysis** ***(N*** **= 152).** (PDF 260 kb)
Additional file 2: **Heat map of 69 genes.** (PDF 255 kb)
Additional file 3: **Seventeen Candidate genes [**11**,** 29**–**44**].** (PDF 271 kb)
Additional file 4: **Result of** **co-expression** **analysis of 69 genes.** (PDF 230 kb)

## Abbreviations

TNIK: Traf2- and Nck- interacting kinase
CRC: Colorectal cancer
IHC: Immunohistochemistry
LCM: Laser capture microdissection
GEO: the Gene Expression Omnibus
RFS: Relapse-free survival
OS: Overall survival
ROC: Receiver operating characteristic
CEA: Carcinoembryonic antigen
TCF4: T-cell for factor 4
LEF: Lymphoid enhancer factor
