# Supplementary material for: Prognostic significance of Traf2- and Nck- interacting kinase (TNIK) in colorectal cancer
Source: BMC Cancer. 2015 Oct 24;15:794. doi: 10.1186/s12885-015-1783-y (PMC4619995; doi:10.1186/s12885-015-1783-y)
Supplement: Additional file 1: — Patients’ characteristics for microarray analysis (N = 152). (PDF 260 kb) [file 12885_2015_1783_MOESM1_ESM.pdf]

Additional file 1: Patients' characteristics for microarray analysis (N = 152)

Variables		No (%)
Age	< 70	86 (57)
	≥ 70	66 (43)
Gender	male	101 (66)
	female	51 (34)
Histology (TNM 7th)	G1	58 (38)
	G2	87 (57)
	G3	7 (5)
Tumor location	right-sided colon	41 (27)
	left-sided colon	76 (50)
	rectum	35 (23)
Stage (TNM 7th)	I	27 (18)
	II	69 (45)
	III	56 (37)
Depth of tumor invasion (TNM 7th)	T1	5 (3)
	T2	28 (18)
	T3	76 (50)
	T4	43 (29)
Lymphatic invasion	(-)	60 (39)
	(+)	92 (61)
Venous invasion	(-)	26 (17)
	(+)	126 (83)
Lymph node metastasis	(-)	96 (63)
	(+)	56 (37)
CEA	< 5 ng/ml	94 (62)
	≥ 5 ng/ml	58 (38)
Distant recurrence	(-)	126 (83)
	(+)	26 (17)
