# Supplementary figures and images for: Prognostic significance of Traf2- and Nck- interacting kinase (TNIK) in colorectal cancer
Source: BMC Cancer. 2015 Oct 24;15:794. doi: 10.1186/s12885-015-1783-y (PMC4619995; doi:10.1186/s12885-015-1783-y)

## Additional file 2: Heat map of 69 genes

Relative expression level

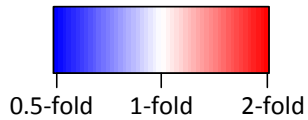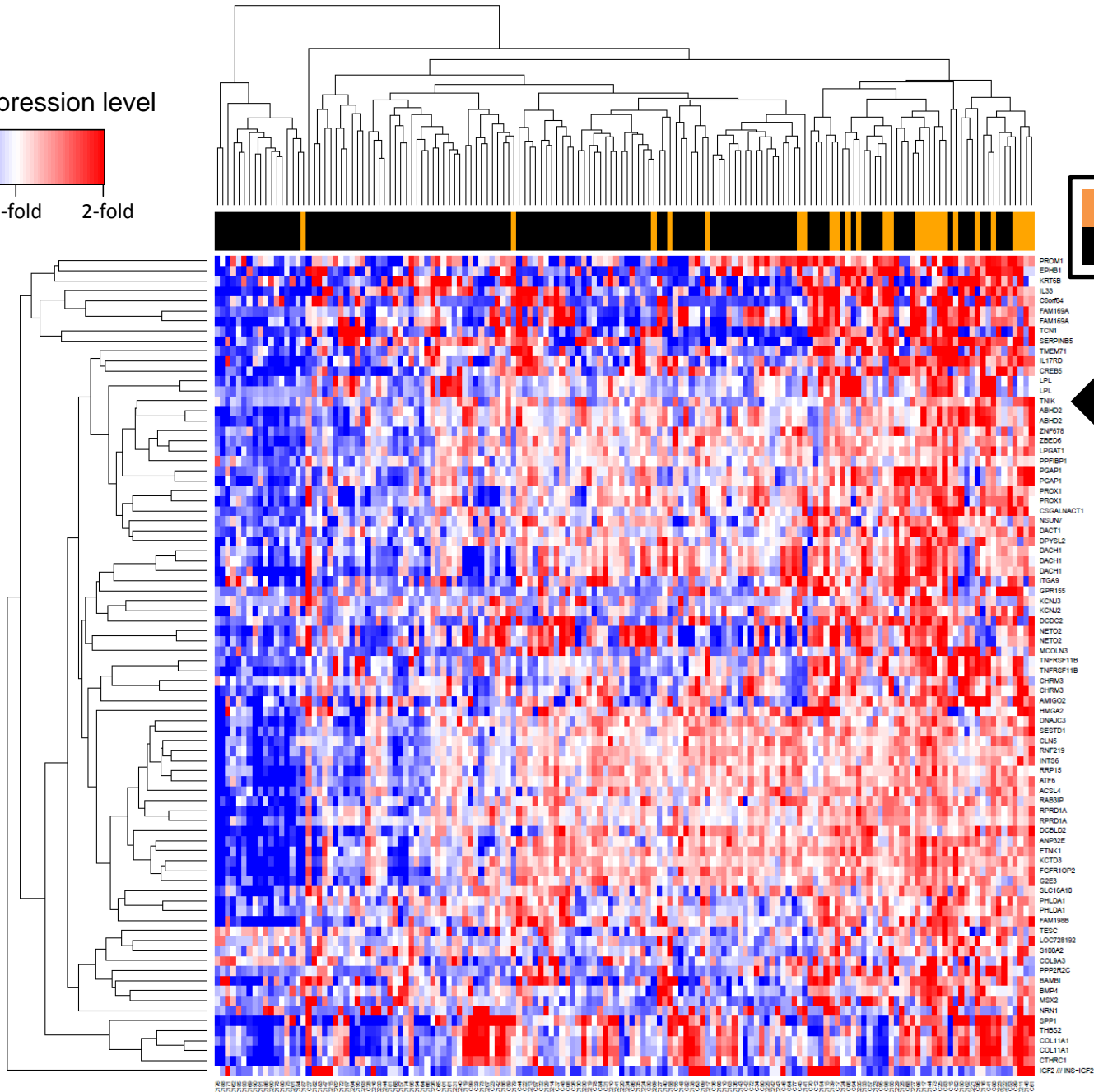

Recurrence  
Non recurrence

TNIK

Supplement: Additional file 2: — Heat map of 69 genes. (PDF 255 kb) [file 12885_2015_1783_MOESM2_ESM.pdf]

#### Additional file 4: Result of co-expression analysis of 69 genes

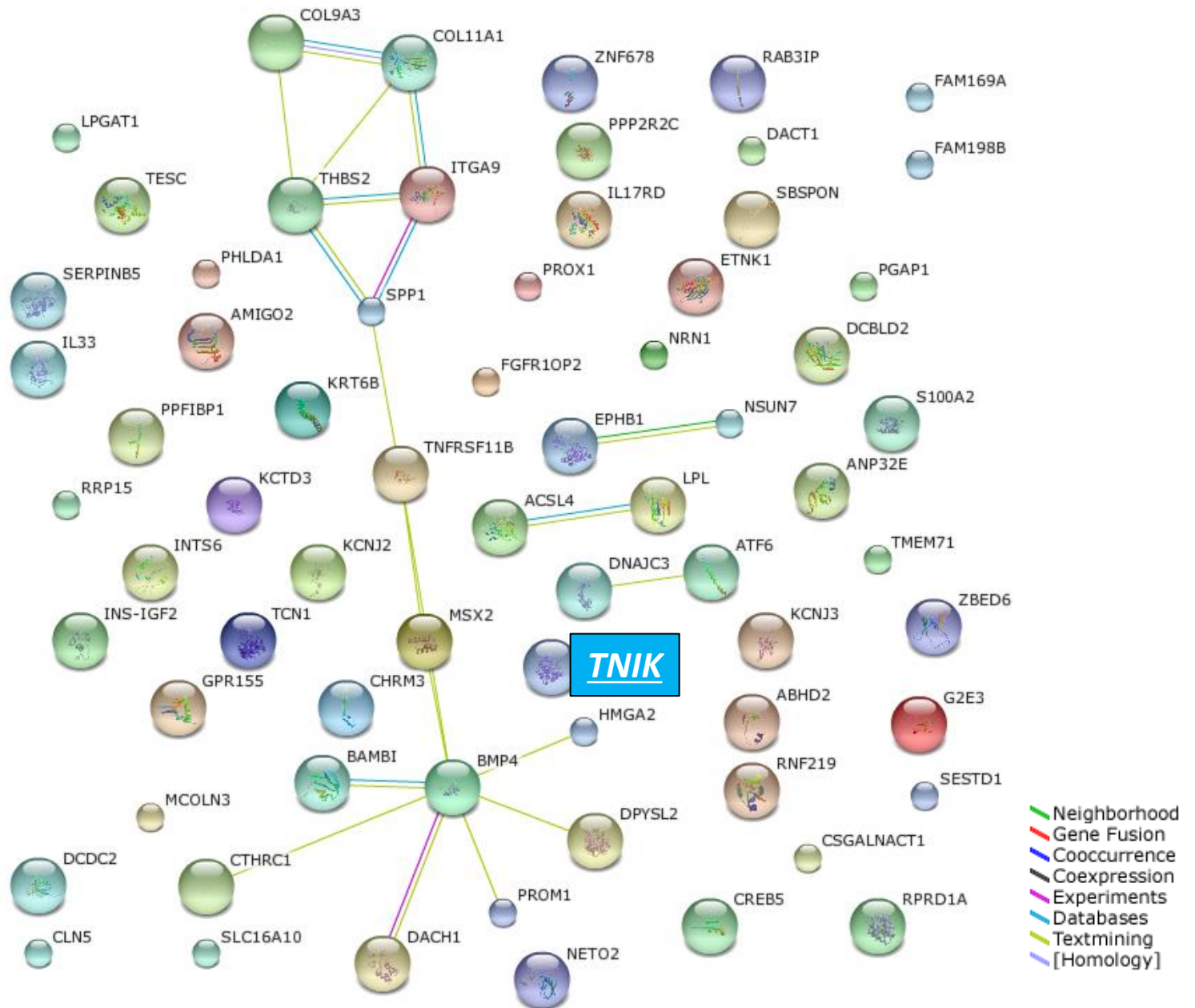

Supplement: Additional file 4: — Result of co-expression analysis of 69 genes. (PDF 230 kb) [file 12885_2015_1783_MOESM4_ESM.pdf]
