# Supplementary material for: Prognostic significance of Traf2- and Nck- interacting kinase (TNIK) in colorectal cancer
Source: BMC Cancer. 2015 Oct 24;15:794. doi: 10.1186/s12885-015-1783-y (PMC4619995; doi:10.1186/s12885-015-1783-y)
Supplement: Additional file 3: — Seventeen Candidate genes [11, 29–44]. (PDF 271 kb) [file 12885_2015_1783_MOESM3_ESM.pdf]

Additional file 3: Seventeen Candidate genes

Gene symbol	Gene title	P value	Fold change	Reported gene character
RPRD1A	regulation of nuclear pre-mRNA domain containing 1A	0.000744	1.68	RPRD1A inhibits the Wnt/β-catenin signaling pathway. (29)
TESC	tescalcin	0.000773	1.71	The EF-hand calcium-binding protein. TESC may interract with NF-κB and colorectal cancer. (30)
ATF6	activating transcription factor 6	0.001125	1.53	A missense polymorphism in ATF6 gene is associated with hepatocellular carcinoma. (31)
ZBED6	zinc finger, BED-type containing 6	0.001207	1.55	Transcriptional modulator ZBED6 affects cell cycle and growth of human colorectal cancer cells. (32)
TNIK	Traf2- and NCK- interacting kinase	0.001370	1.58	TNIK is essential for Wnt signaling and colorectal cancer growth. (11)
PROX1	prospero-related homeobox 1	0.001581	1.75	PROX1 is a specific target of the β-catenin/TCF pathway in the intestinal epithelium. PROX1 is upregulated in colorectal cancer, and may have a role in tumor progression in colorectal cancer. (33)
S100A2	S100 calcium binding protein A2	0.002094	1.79	S100A2 is a predictive biomarker of pancreatic adenocarcinoma. (34)
INTS6	integrator complex subunit 6	0.002243	1.53	Pseudogene INTS6P1 regulates its cognate gene INTS6 through competitive binding of miR-17-5p in hepatocellular carcinoma. (35)
CHRM3	cholinergic receptor, muscarinic 3	0.003200	1.80	CHRM3 is a novel prognostic factor of poor prognosis in patients with endometrial carcinoma. (36)
KCNJ2	potassium inwardly-rectifying channel, subfamily J, member 2	0.003200	1.51	Upregulation of the inwardly rectifying potassium channel Kir2.1 (KCNJ2) modulates multidrug resistance of small-cell lung cancer under the regulation of miR-7 and the Ras/MAPK pathway. (37)
HMGA2	high mobility group AT-hook 2	0.003961	1.88	High-mobility group A2 overexpression is an unfavorable prognostic biomarker for nasopharyngeal carcinoma patients. (38)
BMP4	bone morphogenetic protein 4	0.007427	1.50	BMP4 inhibits breast cancer metastasis by blocking myeloid-derived suppressor cell activity. (39)
PHLDA1	pleckstrin homology-like domain, family A, member 1	0.008633	1.57	PHLDA1 is a crucial negative regulator and effector of Aurora A kinase in breast cancer. (40)
IGF2	insulin-like growth factor 2	0.012791	1.97	High IGF2 expression is associated with poor clinical outcome in human ovarian cancer. (41)
DACH1	dachshund homolog 1	0.020715	1.64	Silencing DACH1 promotes esophageal cancer growth by inhibiting TGF-β signaling. (42)
COL11A1	collagen, type XI, alpha 1	0.039616	1.57	COL11A1 promotes tumor progression and predicts poor clinical outcome in ovarian cancer. (43)
DACT1	dapper, antagonist of beta-catenin, homolog 1	0.041573	1.52	Expression and epigenetic regulation of DACT1 and DACT2 in oral squamous cell carcinoma. (44)
